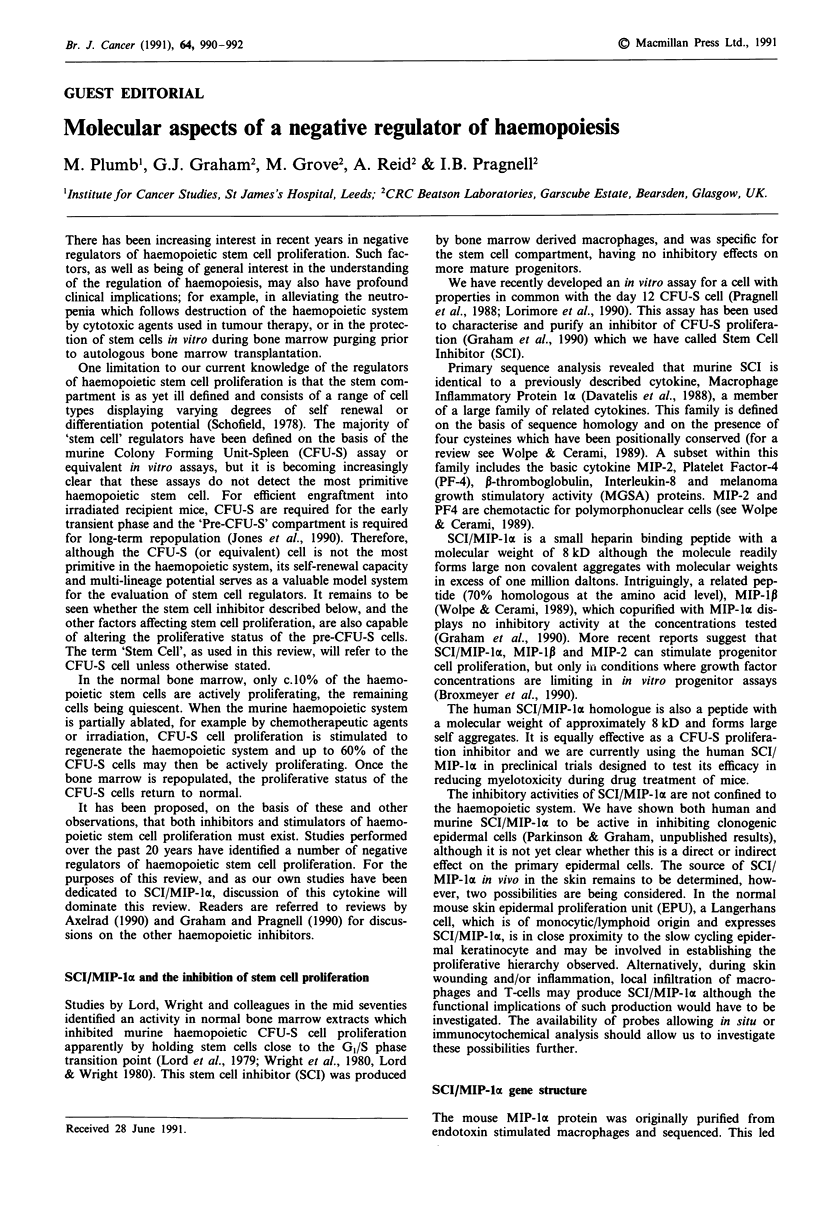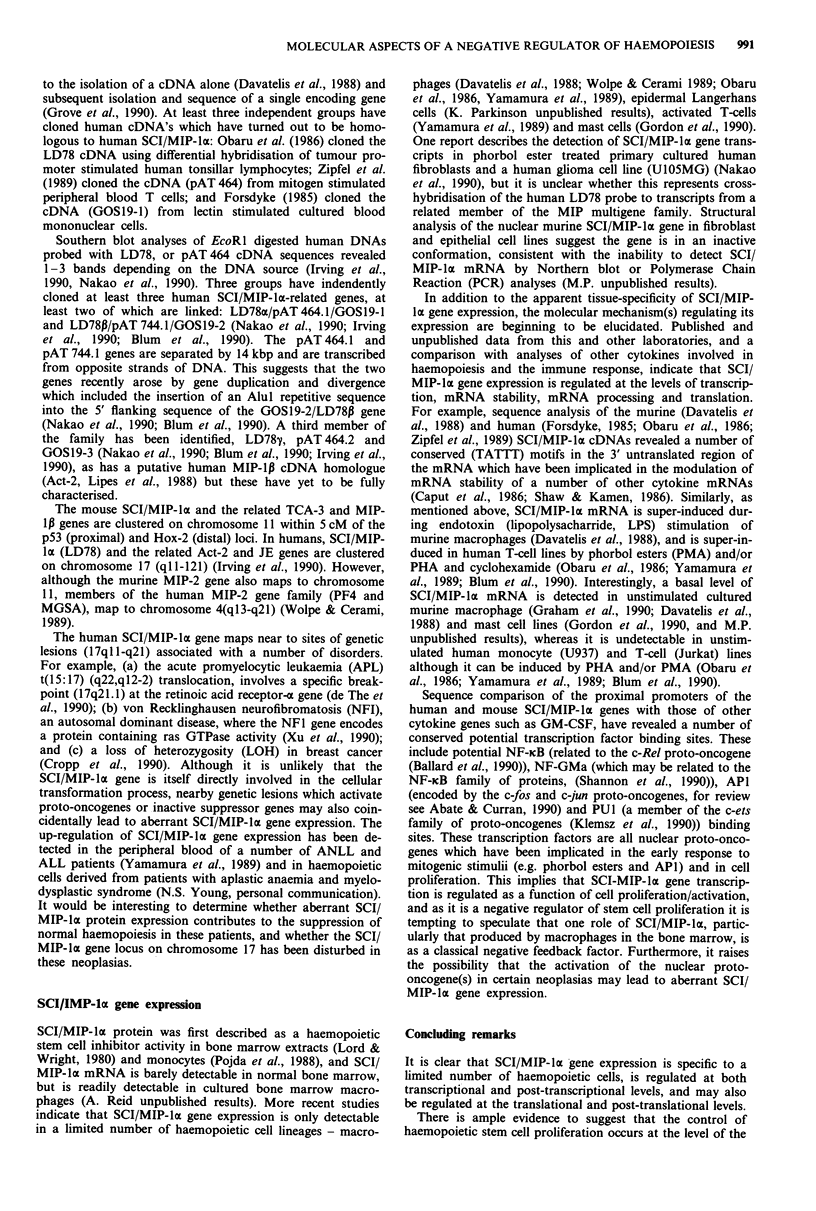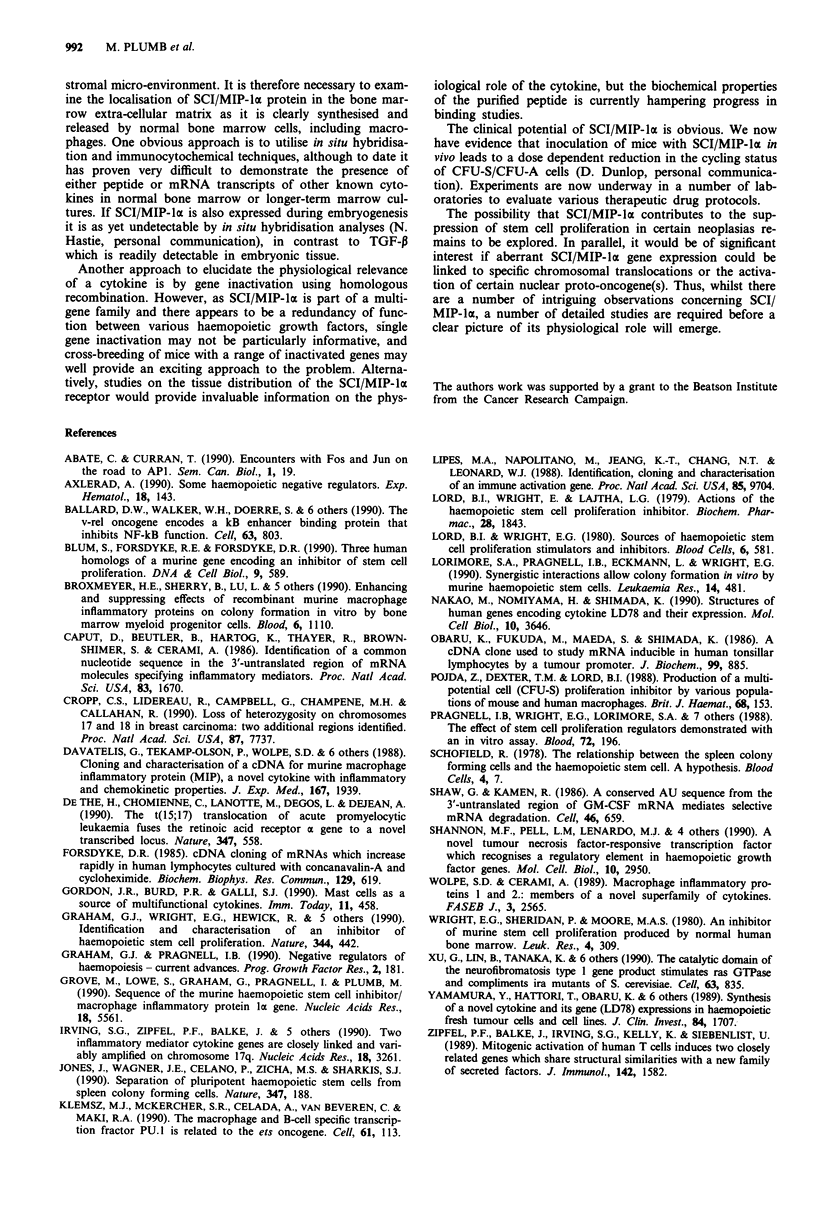# Molecular aspects of a negative regulator of haemopoiesis.

**DOI:** 10.1038/bjc.1991.452

**Published:** 1991-12

**Authors:** M. Plumb, G. J. Graham, M. Grove, A. Reid, I. B. Pragnell


Br. J. Cancer (1991), 64, 990-992 © Macmillan Press Ltd., 1991

GUEST EDITORIAL

### Molecular aspects of a negative regulator of haemopoiesis

M. Plumb[1], G.J. Graham[2], M. Grove[2], A. Reid[2] & I.B. Pragnell[2]

[1]*Institute for Cancer Studies, St James's Hospital, Leeds;* [2]*CRC Beatson Laboratories, Garscube Estate, Bearsden, Glasgow, UK.*

There has been increasing interest in recent years in negative regulators of haemopoietic stem cell proliferation. Such factors, as well as being of general interest in the understanding of the regulation of haemopoiesis, may also have profound clinical implications; for example, in alleviating the neutropenia which follows destruction of the haemopoietic system by cytotoxic agents used in tumour therapy, or in the protection of stem cells *in vitro* during bone marrow purging prior to autologous bone marrow transplantation.

One limitation to our current knowledge of the regulators of haemopoietic stem cell proliferation is that the stem compartment is as yet ill defined and consists of a range of cell types displaying varying degrees of self renewal or differentiation potential (Schofield, 1978). The majority of 'stem cell' regulators have been defined on the basis of the murine Colony Forming Unit-Spleen (CFU-S) assay or equivalent *in vitro* assays, but it is becoming increasingly clear that these assays do not detect the most primitive haemopoietic stem cell. For efficient engraftment into irradiated recipient mice, CFU-S are required for the early transient phase and the 'Pre-CFU-S' compartment is required for long-term repopulation (Jones *et al.*, 1990). Therefore, although the CFU-S (or equivalent) cell is not the most primitive in the haemopoietic system, its self-renewal capacity and multi-lineage potential serves as a valuable model system for the evaluation of stem cell regulators. It remains to be seen whether the stem cell inhibitor described below, and the other factors affecting stem cell proliferation, are also capable of altering the proliferative status of the pre-CFU-S cells. The term 'Stem Cell', as used in this review, will refer to the CFU-S cell unless otherwise stated.

In the normal bone marrow, only c.10% of the haemopoietic stem cells are actively proliferating, the remaining cells being quiescent. When the murine haemopoietic system is partially ablated, for example by chemotherapeutic agents or irradiation, CFU-S cell proliferation is stimulated to regenerate the haemopoietic system and up to 60% of the CFU-S cells may then be actively proliferating. Once the bone marrow is repopulated, the proliferative status of the CFU-S cells return to normal.

It has been proposed, on the basis of these and other observations, that both inhibitors and stimulators of haemopoietic stem cell proliferation must exist. Studies performed over the past 20 years have identified a number of negative regulators of haemopoietic stem cell proliferation. For the purposes of this review, and as our own studies have been dedicated to SCI/MIP-1α, discussion of this cytokine will dominate this review. Readers are referred to reviews by Axelrad (1990) and Graham and Pragnell (1990) for discussions on the other haemopoietic inhibitors.

### SCI/MIP-1α and the inhibition of stem cell proliferation

Studies by Lord, Wright and colleagues in the mid seventies identified an activity in normal bone marrow extracts which inhibited murine haemopoietic CFU-S cell proliferation apparently by holding stem cells close to the $G_1/S$ phase transition point (Lord *et al.*, 1979; Wright *et al.*, 1980, Lord & Wright 1980). This stem cell inhibitor (SCI) was produced by bone marrow derived macrophages, and was specific for the stem cell compartment, having no inhibitory effects on more mature progenitors.

We have recently developed an *in vitro* assay for a cell with properties in common with the day 12 CFU-S cell (Pragnell *et al.*, 1988; Lorimore *et al.*, 1990). This assay has been used to characterise and purify an inhibitor of CFU-S proliferation (Graham *et al.*, 1990) which we have called Stem Cell Inhibitor (SCI).

Primary sequence analysis revealed that murine SCI is identical to a previously described cytokine, Macrophage Inflammatory Protein 1α (Davatelis *et al.*, 1988), a member of a large family of related cytokines. This family is defined on the basis of sequence homology and on the presence of four cysteines which have been positionally conserved (for a review see Wolpe & Cerami, 1989). A subset within this family includes the basic cytokine MIP-2, Platelet Factor-4 (PF-4), β-thromboglobulin, Interleukin-8 and melanoma growth stimulatory activity (MGSA) proteins. MIP-2 and PF4 are chemotactic for polymorphonuclear cells (see Wolpe & Cerami, 1989).

SCI/MIP-1α is a small heparin binding peptide with a molecular weight of 8 kD although the molecule readily forms large non covalent aggregates with molecular weights in excess of one million daltons. Intriguingly, a related peptide (70% homologous at the amino acid level), MIP-1β (Wolpe & Cerami, 1989), which copurified with MIP-1α displays no inhibitory activity at the concentrations tested (Graham *et al.*, 1990). More recent reports suggest that SCI/MIP-1α, MIP-1β and MIP-2 can stimulate progenitor cell proliferation, but only in conditions where growth factor concentrations are limiting in *in vitro* progenitor assays (Broxmeyer *et al.*, 1990).

The human SCI/MIP-1α homologue is also a peptide with a molecular weight of approximately 8 kD and forms large self aggregates. It is equally effective as a CFU-S proliferation inhibitor and we are currently using the human SCI/MIP-1α in preclinical trials designed to test its efficacy in reducing myelotoxicity during drug treatment of mice.

The inhibitory activities of SCI/MIP-1α are not confined to the haemopoietic system. We have shown both human and murine SCI/MIP-1α to be active in inhibiting clonogenic epidermal cells (Parkinson & Graham, unpublished results), although it is not yet clear whether this is a direct or indirect effect on the primary epidermal cells. The source of SCI/MIP-1α *in vivo* in the skin remains to be determined, however, two possibilities are being considered. In the normal mouse skin epidermal proliferation unit (EPU), a Langerhans cell, which is of monocytic/lymphoid origin and expresses SCI/MIP-1α, is in close proximity to the slow cycling epidermal keratinocyte and may be involved in establishing the proliferative hierarchy observed. Alternatively, during skin wounding and/or inflammation, local infiltration of macrophages and T-cells may produce SCI/MIP-1α although the functional implications of such production would have to be investigated. The availability of probes allowing *in situ* or immunocytochemical analysis should allow us to investigate these possibilities further.

### SCI/MIP-1α gene structure

The mouse MIP-1α protein was originally purified from endotoxin stimulated macrophages and sequenced. This led

Received 28 June 1991.

to the isolation of a cDNA alone (Davatelis *et al.*, 1988) and subsequent isolation and sequence of a single encoding gene (Grove *et al.*, 1990). At least three independent groups have cloned human cDNA's which have turned out to be homologous to human SCI/MIP-1α: Obaru *et al.* (1986) cloned the LD78 cDNA using differential hybridisation of tumour promoter stimulated human tonsillar lymphocytes; Zipfel *et al.* (1989) cloned the cDNA (pAT 464) from mitogen stimulated peripheral blood T cells; and Forsdyke (1985) cloned the cDNA (GOS19-1) from lectin stimulated cultured blood mononuclear cells.

Southern blot analyses of *Eco*R1 digested human DNAs probed with LD78, or pAT 464 cDNA sequences revealed 1–3 bands depending on the DNA source (Irving *et al.*, 1990, Nakao *et al.*, 1990). Three groups have indendently cloned at least three human SCI/MIP-1α-related genes, at least two of which are linked: LD78α/pAT 464.1/GOS19-1 and LD78β/pAT 744.1/GOS19-2 (Nakao *et al.*, 1990; Irving *et al.*, 1990; Blum *et al.*, 1990). The pAT 464.1 and pAT 744.1 genes are separated by 14 kbp and are transcribed from opposite strands of DNA. This suggests that the two genes recently arose by gene duplication and divergence which included the insertion of an Alul repetitive sequence into the 5′ flanking sequence of the GOS19-2/LD78β gene (Nakao *et al.*, 1990; Blum *et al.*, 1990). A third member of the family has been identified, LD78γ, pAT 464.2 and GOS19-3 (Nakao *et al.*, 1990; Blum *et al.*, 1990; Irving *et al.*, 1990), as has a putative human MIP-1β cDNA homologue (Act-2, Lipes *et al.*, 1988) but these have yet to be fully characterised.

The mouse SCI/MIP-1α and the related TCA-3 and MIP-1β genes are clustered on chromosome 11 within 5 cM of the p53 (proximal) and Hox-2 (distal) loci. In humans, SCI/MIP-1α (LD78) and the related Act-2 and JE genes are clustered on chromosome 17 (q11-121) (Irving *et al.*, 1990). However, although the murine MIP-2 gene also maps to chromosome 11, members of the human MIP-2 gene family (PF4 and MGSA), map to chromosome 4(q13-q21) (Wolpe & Cerami, 1989).

The human SCI/MIP-1α gene maps near to sites of genetic lesions (17q11-q21) associated with a number of disorders. For example, (a) the acute promyelocytic leukaemia (APL) t(15:17) (q22,q12-2) translocation, involves a specific breakpoint (17q21.1) at the retinoic acid receptor-α gene (de The *et al.*, 1990); (b) von Recklinghausen neurofibromatosis (NFI), an autosomal dominant disease, where the NF1 gene encodes a protein containing ras GTPase activity (Xu *et al.*, 1990); and (c) a loss of heterozygosity (LOH) in breast cancer (Cropp *et al.*, 1990). Although it is unlikely that the SCI/MIP-1α gene is itself directly involved in the cellular transformation process, nearby genetic lesions which activate proto-oncogenes or inactive suppressor genes may also coincidentally lead to aberrant SCI/MIP-1α gene expression. The up-regulation of SCI/MIP-1α gene expression has been detected in the peripheral blood of a number of ANLL and ALL patients (Yamamura *et al.*, 1989) and in haemopoietic cells derived from patients with aplastic anaemia and myelodysplastic syndrome (N.S. Young, personal communication). It would be interesting to determine whether aberrant SCI/MIP-1α protein expression contributes to the suppression of normal haemopoiesis in these patients, and whether the SCI/MIP-1α gene locus on chromosome 17 has been disturbed in these neoplasias.

### SCI/IMP-1α gene expression

SCI/MIP-1α protein was first described as a haemopoietic stem cell inhibitor activity in bone marrow extracts (Lord & Wright, 1980) and monocytes (Pojda *et al.*, 1988), and SCI/MIP-1α mRNA is barely detectable in normal bone marrow, but is readily detectable in cultured bone marrow macrophages (A. Reid unpublished results). More recent studies indicate that SCI/MIP-1α gene expression is only detectable in a limited number of haemopoietic cell lineages – macrophages (Davatelis *et al.*, 1988; Wolpe & Cerami 1989; Obaru *et al.*, 1986, Yamamura *et al.*, 1989), epidermal Langerhans cells (K. Parkinson unpublished results), activated T-cells (Yamamura *et al.*, 1989) and mast cells (Gordon *et al.*, 1990). One report describes the detection of SCI/MIP-1α gene transcripts in phorbol ester treated primary cultured human fibroblasts and a human glioma cell line (U105MG) (Nakao *et al.*, 1990), but it is unclear whether this represents cross-hybridisation of the human LD78 probe to transcripts from a related member of the MIP multigene family. Structural analysis of the nuclear murine SCI/MIP-1α gene in fibroblast and epithelial cell lines suggest the gene is in an inactive conformation, consistent with the inability to detect SCI/MIP-1α mRNA by Northern blot or Polymerase Chain Reaction (PCR) analyses (M.P. unpublished results).

In addition to the apparent tissue-specificity of SCI/MIP-1α gene expression, the molecular mechanism(s) regulating its expression are beginning to be elucidated. Published and unpublished data from this and other laboratories, and a comparison with analyses of other cytokines involved in haemopoiesis and the immune response, indicate that SCI/MIP-1α gene expression is regulated at the levels of transcription, mRNA stability, mRNA processing and translation. For example, sequence analysis of the murine (Davatelis *et al.*, 1988) and human (Forsdyke, 1985; Obaru *et al.*, 1986; Zipfel *et al.*, 1989) SCI/MIP-1α cDNAs revealed a number of conserved (TATTT) motifs in the 3′ untranslated region of the mRNA which have been implicated in the modulation of mRNA stability of a number of other cytokine mRNAs (Caput *et al.*, 1986; Shaw & Kamen, 1986). Similarly, as mentioned above, SCI/MIP-1α mRNA is super-induced during endotoxin (lipopolysacharride, LPS) stimulation of murine macrophages (Davatelis *et al.*, 1988), and is super-induced in human T-cell lines by phorbol esters (PMA) and/or PHA and cyclohexamide (Obaru *et al.*, 1986; Yamamura *et al.*, 1989; Blum *et al.*, 1990). Interestingly, a basal level of SCI/MIP-1α mRNA is detected in unstimulated cultured murine macrophage (Graham *et al.*, 1990; Davatelis *et al.*, 1988) and mast cell lines (Gordon *et al.*, 1990, and M.P. unpublished results), whereas it is undetectable in unstimulated human monocyte (U937) and T-cell (Jurkat) lines although it can be induced by PHA and/or PMA (Obaru *et al.*, 1986; Yamamura *et al.*, 1989; Blum *et al.*, 1990).

Sequence comparison of the proximal promoters of the human and mouse SCI/MIP-1α genes with those of other cytokine genes such as GM-CSF, have revealed a number of conserved potential transcription factor binding sites. These include potential NF-κB (related to the c-*Rel* proto-oncogene (Ballard *et al.*, 1990)), NF-GMa (which may be related to the NF-κB family of proteins, (Shannon *et al.*, 1990)), AP1 (encoded by the c-*fos* and c-*jun* proto-oncogenes, for review see Abate & Curran, 1990) and PU1 (a member of the c-*ets* family of proto-oncogenes (Klemsz *et al.*, 1990)) binding sites. These transcription factors are all nuclear proto-oncogenes which have been implicated in the early response to mitogenic stimulii (e.g. phorbol esters and AP1) and in cell proliferation. This implies that SCI-MIP-1α gene transcription is regulated as a function of cell proliferation/activation, and as it is a negative regulator of stem cell proliferation it is tempting to speculate that one role of SCI/MIP-1α, particularly that produced by macrophages in the bone marrow, is as a classical negative feedback factor. Furthermore, it raises the possibility that the activation of the nuclear proto-oncogene(s) in certain neoplasias may lead to aberrant SCI/MIP-1α gene expression.

### Concluding remarks

It is clear that SCI/MIP-1α gene expression is specific to a limited number of haemopoietic cells, is regulated at both transcriptional and post-transcriptional levels, and may also be regulated at the translational and post-translational levels.

There is ample evidence to suggest that the control of haemopoietic stem cell proliferation occurs at the level of the stromal micro-environment. It is therefore necessary to examine the localisation of SCI/MIP-1α protein in the bone marrow extra-cellular matrix as it is clearly synthesised and released by normal bone marrow cells, including macrophages. One obvious approach is to utilise *in situ* hybridisation and immunocytochemical techniques, although to date it has proven very difficult to demonstrate the presence of either peptide or mRNA transcripts of other known cytokines in normal bone marrow or longer-term marrow cultures. If SCI/MIP-1α is also expressed during embryogenesis it is as yet undetectable by *in situ* hybridisation analyses (N. Hastie, personal communication), in contrast to TGF-β which is readily detectable in embryonic tissue.

Another approach to elucidate the physiological relevance of a cytokine is by gene inactivation using homologous recombination. However, as SCI/MIP-1α is part of a multigene family and there appears to be a redundancy of function between various haemopoietic growth factors, single gene inactivation may not be particularly informative, and cross-breeding of mice with a range of inactivated genes may well provide an exciting approach to the problem. Alternatively, studies on the tissue distribution of the SCI/MIP-1α receptor would provide invaluable information on the physiological role of the cytokine, but the biochemical properties of the purified peptide is currently hampering progress in binding studies.

The clinical potential of SCI/MIP-1α is obvious. We now have evidence that inoculation of mice with SCI/MIP-1α *in vivo* leads to a dose dependent reduction in the cycling status of CFU-S/CFU-A cells (D. Dunlop, personal communication). Experiments are now underway in a number of laboratories to evaluate various therapeutic drug protocols.

The possibility that SCI/MIP-1α contributes to the suppression of stem cell proliferation in certain neoplasias remains to be explored. In parallel, it would be of significant interest if aberrant SCI/MIP-1α gene expression could be linked to specific chromosomal translocations or the activation of certain nuclear proto-oncogene(s). Thus, whilst there are a number of intriguing observations concerning SCI/MIP-1α, a number of detailed studies are required before a clear picture of its physiological role will emerge.

The authors work was supported by a grant to the Beatson Institute from the Cancer Research Campaign.

### References

ABATE, C. & CURRAN, T. (1990). Encounters with Fos and Jun on the road to AP1. *Sem. Can. Biol.*, **1**, 19.

AXLERAD, A. (1990). Some haemopoietic negative regulators. *Exp. Hematol.*, **18**, 143.

BALLARD, D.W., WALKER, W.H., DOERRE, S. & 6 others (1990). The v-rel oncogene encodes a kB enhancer binding protein that inhibits NF-kB function. *Cell*, **63**, 803.

BLUM, S., FORSDYKE, R.E. & FORSDYKE, D.R. (1990). Three human homologs of a murine gene encoding an inhibitor of stem cell proliferation. *DNA & Cell Biol.*, **9**, 589.

BROXMEYER, H.E., SHERRY, B., LU, L. & 5 others (1990). Enhancing and suppressing effects of recombinant murine macrophage inflammatory proteins on colony formation in vitro by bone marrow myeloid progenitor cells. *Blood*, **6**, 1110.

CAPUT, D., BEUTLER, B., HARTOG, K., THAYER, R., BROWN-SHIMER, S. & CERAMI, A. (1986). Identification of a common nucleotide sequence in the 3′-untranslated region of mRNA molecules specifying inflammatory mediators. *Proc. Natl Acad. Sci. USA*, **83**, 1670.

CROPP, C.S., LIDEREAU, R., CAMPBELL, G., CHAMPENE, M.H. & CALLAHAN, R. (1990). Loss of heterozygosity on chromosomes 17 and 18 in breast carcinoma: two additional regions identified. *Proc. Natl Acad. Sci. USA*, **87**, 7737.

DAVATELIS, G., TEKAMP-OLSON, P., WOLPE, S.D. & 6 others (1988). Cloning and characterisation of a cDNA for murine macrophage inflammatory protein (MIP), a novel cytokine with inflammatory and chemokinetic properties. *J. Exp. Med.*, **167**, 1939.

DE THE, H., CHOMIENNE, C., LANOTTE, M., DEGOS, L. & DEJEAN, A. (1990). The t(15;17) translocation of acute promyelocytic leukaemia fuses the retinoic acid receptor α gene to a novel transcribed locus. *Nature*, **347**, 558.

FORSDYKE, D.R. (1985). cDNA cloning of mRNAs which increase rapidly in human lymphocytes cultured with concanavalin-A and cycloheximide. *Biochem. Biophys. Res. Commun.*, **129**, 619.

GORDON, J.R., BURD, P.R. & GALLI, S.J. (1990). Mast cells as a source of multifunctional cytokines. *Imm. Today*, **11**, 458.

GRAHAM, G.J., WRIGHT, E.G., HEWICK, R. & 5 others (1990). Identification and characterisation of an inhibitor of haemopoietic stem cell proliferation. *Nature*, **344**, 442.

GRAHAM, G.J. & PRAGNELL, I.B. (1990). Negative regulators of haemopoiesis – current advances. *Prog. Growth Factor Res.*, **2**, 181.

GROVE, M., LOWE, S., GRAHAM, G., PRAGNELL, I. & PLUMB, M. (1990). Sequence of the murine haemopoietic stem cell inhibitor/macrophage inflammatory protein 1α gene. *Nucleic Acids Res.*, **18**, 5561.

IRVING, S.G., ZIPFEL, P.F., BALKE, J. & 5 others (1990). Two inflammatory mediator cytokine genes are closely linked and variably amplified on chromosome 17q. *Nucleic Acids Res.*, **18**, 3261.

JONES, J., WAGNER, J.E., CELANO, P., ZICHA, M.S. & SHARKIS, S.J. (1990). Separation of pluripotent haemopoietic stem cells from spleen colony forming cells. *Nature*, **347**, 188.

KLEMSZ, M.J., MCKERCHER, S.R., CELADA, A., VAN BEVEREN, C. & MAKI, R.A. (1990). The macrophage and B-cell specific transcription fraction PU.1 is related to the *ets* oncogene. *Cell*, **61**, 113.

LIPES, M.A., NAPOLITANO, M., JEANG, K.-T., CHANG, N.T. & LEONARD, W.J. (1988). Identification, cloning and characterisation of an immune activation gene. *Proc. Natl Acad. Sci. USA*, **85**, 9704.

LORD, B.I., WRIGHT, E. & LAJTHA, L.G. (1979). Actions of the haemopoietic stem cell proliferation inhibitor. *Biochem. Pharmac.*, **28**, 1843.

LORD, B.I. & WRIGHT, E.G. (1980). Sources of haemopoietic stem cell proliferation stimulators and inhibitors. *Blood Cells*, **6**, 581.

LORIMORE, S.A., PRAGNELL, I.B., ECKMANN, L. & WRIGHT, E.G. (1990). Synergistic interactions allow colony formation *in vitro* by murine haemopoietic stem cells. *Leukaemia Res.*, **14**, 481.

NAKAO, M., NOMIYAMA, H. & SHIMADA, K. (1990). Structures of human genes encoding cytokine LD78 and their expression. *Mol. Cell Biol.*, **10**, 3646.

OBARU, K., FUKUDA, M., MAEDA, S. & SHIMADA, K. (1986). A cDNA clone used to study mRNA inducible in human tonsillar lymphocytes by a tumour promoter. *J. Biochem.*, **99**, 885.

POJDA, Z., DEXTER, T.M. & LORD, B.I. (1988). Production of a multipotential cell (CFU-S) proliferation inhibitor by various populations of mouse and human macrophages. *Brit. J. Haemat.*, **68**, 153.

PRAGNELL, I.B, WRIGHT, E.G., LORIMORE, S.A. & 7 others (1988). The effect of stem cell proliferation regulators demonstrated with an in vitro assay. *Blood*, **72**, 196.

SCHOFIELD, R. (1978). The relationship between the spleen colony forming cells and the haemopoietic stem cell. A hypothesis. *Blood Cells*, **4**, 7.

SHAW, G. & KAMEN, R. (1986). A conserved AU sequence from the 3′-untranslated region of GM-CSF mRNA mediates selective mRNA degradation. *Cell*, **46**, 659.

SHANNON, M.F., PELL, L.M, LENARDO, M.J. & 4 others (1990). A novel tumour necrosis factor-responsive transcription factor which recognises a regulatory element in haemopoietic growth factor genes. *Mol. Cell. Biol.*, **10**, 2950.

WOLPE, S.D. & CERAMI, A. (1989). Macrophage inflammatory proteins 1 and 2.: members of a novel superfamily of cytokines. *FASEB J.*, **3**, 2565.

WRIGHT, E.G., SHERIDAN, P. & MOORE, M.A.S. (1980). An inhibitor of murine stem cell proliferation produced by normal human bone marrow. *Leuk. Res.*, **4**, 309.

XU, G., LIN, B., TANAKA, K. & 6 others (1990). The catalytic domain of the neurofibromatosis type 1 gene product stimulates ras GTPase and compliments ira mutants of S. cerevisiae. *Cell*, **63**, 835.

YAMAMURA, Y., HATTORI, T., OBARU, K. & 6 others (1989). Synthesis of a novel cytokine and its gene (LD78) expressions in haemopoietic fresh tumour cells and cell lines. *J. Clin. Invest.*, **84**, 1707.

ZIPFEL, P.F., BALKE, J., IRVING, S.G., KELLY, K. & SIEBENLIST, U. (1989). Mitogenic activation of human T cells induces two closely related genes which share structural similarities with a new family of secreted factors. *J. Immunol.*, **142**, 1582.